# Percutaneous Interventricular Septal Access Guided by Subcostal Echocardiography and Fluoroscopy for Ventricular Tachycardia Ablation in a Patient with Aortic and Mitral Mechanical Valves

**DOI:** 10.19102/icrm.2019.100702

**Published:** 2019-07-15

**Authors:** Dursun Aras, Serkan Topaloglu, Ozcan Ozeke, Firat Ozcan, Serkan Cay, Zehra Golbasi

**Affiliations:** ^1^Department of Cardiology, Turkiye Yuksek Ihtisas Training and Research Hospital, Health Sciences University, Ankara, Turkey

**Keywords:** Ablation, aortic and mitral mechanical valves, double mechanical valve, transventricular septal access, ventricular tachycardia

## Abstract

Mechanical prosthetic aortic and mitral valves preclude either a retrograde aortic or transseptal approach to the left ventricular (LV) endocardium. Several operators have reported on the application of nonconventional techniques for ventricular tachycardia (VT) ablation including transventricular septal puncture, epicardial approach, transmechanical valve approach, transcoronary venous approach, and transapical approach. Incorporating transventricular access to the LV under intracardiac echocardiography (ICE) guidance has been previously attempted in VT ablation procedures in patients with both aortic and mitral mechanical valves. However, while ICE is readily used in the United States, its use is less common in Europe, since the health insurance agencies largely do not cover the costs of ICE catheters. We therefore herein present a case of VT ablation in the LV using a transventricular approach in a patient who underwent mechanical double valve replacement performed under subcostal echocardiographic and fluoroscopic guidance.

## Introduction

Mechanical prosthetic aortic and mitral valves preclude either a retrograde aortic or transseptal approach to the left ventricular (LV) endocardium. Several operators have reported previously on the use of nonconventional techniques during ventricular tachycardia (VT) ablation such as transventricular septal puncture,^1,2^ epicardial approach,^3,4^ transmechanical valve approach,^5^ transcoronary venous approach,^6^ or transapical approach.^7,8^ We present a case of transventricular VT ablation in a patient with electrical storm and mechanical double valve replacement.

## Case report

**Figure 1:**
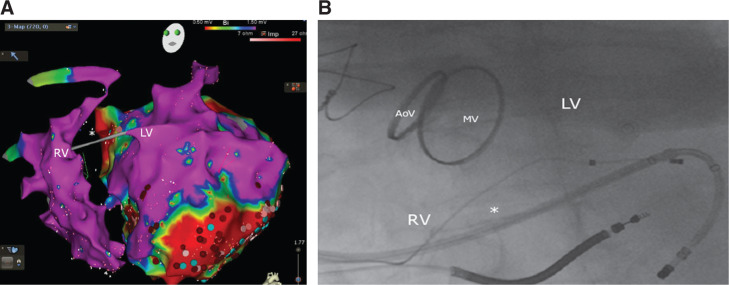
**A:** Left anterior oblique image obtained using the CARTO^®^ 3 three-dimensional electroanatomic mapping system (Biosense Webster, Diamond Bar, CA, USA). **B:** Right anterior oblique image obtained using the fluoroscopy shows the transventricular crossing of a 3.5-mm irrigated-tip ablation catheter (*). An extensive area of low bipolar voltage (< 1.5 mV) consistent with the scar was present in the inferolateral LV apex extending to the accessible mid-LV **(A)**.

**Figure 2:**
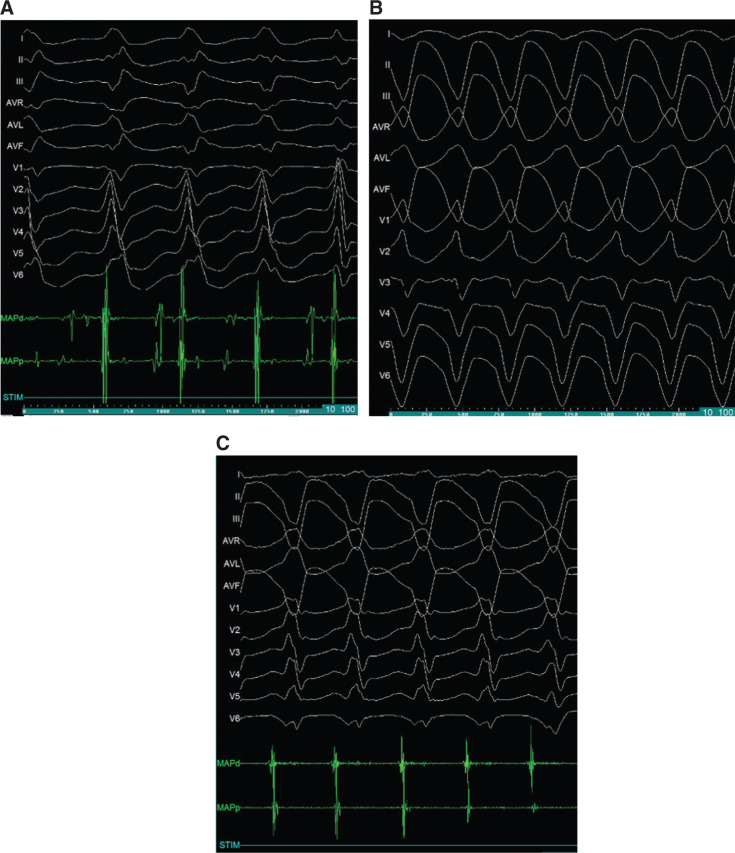
**A, B, and C:** Three different tachycardia morphologies induced and the areas of significant middiastolic potentials during ventricular tachycardia **(A and C)** ablated. AoV: prosthetic aortic valve; LV: left ventricle; MV: prosthetic mitral valve; RV: right ventricle.

A 69-year-old male patient with nonischemic dilated cardiomyopathy was referred for VT ablation due to electrical storm. He had a history of double valve replacement and biventricular pacemaker implantation. Since his documented VT morphologies were compatible with the LV posterobasal and apical regions, we chose to perform transventricular crossing, as it was thought that it could be potentially difficult to reach all parts of the LV apex using transapical access. The right internal jugular vein was accessed, and a Swartz™ Braided SL1 Transseptal Guiding Introducer Sheath (Cardion, Brno, the Czech Republic) was advanced to the basal right ventricular septum. We preemptively prepared the Amplatzer™ Muscular Ventricular Septal Defect Occluder device (Abbott Laboratories, Chicago, IL, USA) as a bailout plan for use if there was a catastrophic septal defect at the time of removal. After confirming there was a safe distance from the coronary septal perforator branches with both left and right coronary angiography and no entrapment of the tricuspid septal leaflet by subcostal echocardiography **(Figures 1A and 1B and Videos 1 and 2)**, the interventricular septum was crossed with a Brockenbrough needle under uninterrupted warfarin and intravenous heparin therapy. It was difficult to dilate and advance the aforementioned Swartz™ Braided SL1 sheath (Cardion, Brno, the Czech Republic) from the transventricular septum; however, the catheter manipulation was easy to perform after the crossing was complete. The three different VTs originating from the LV apical and posterobasal regions were induced **(Figures 2A and 2B and Video 3)** and late potential substrate ablation during sinus rhythm and the areas of the middiastolic potentials during VT **(Figures 2A and 2C)** were ablated. In total, the procedure lasted about 5.3 hours without intraprocedural complications or residual ventricular septal defect **(Video 4)**. He recovered uneventfully and experienced one VT episode that responded to antitachycardia pacing therapy one day later. He was discharged five days after the procedure with heart failure therapy (ie, metoprolol, aldactone, furosemide, ramipril) and mexiletine. At present, this patient is being followed up with in the outpatient clinic.

## Discussion

In patients with mechanical double valve replacement and VT, catheter ablation may be prevented by limited access to the LV. However, direct access to the LV cavity by way of a percutaneous LV apical puncture through the intercostal space overlying the apex or through a left minithoracotomy or left lateral thoracotomy is a viable option for the mapping and ablation of LV VTs.^4,8^ Although epicardial VT ablation is a potentially useful method in patients with mechanical aortic and mitral valves,^9^ the coronary venous system approach^6^ or transventricular septal access^1,2^ have additionally been applied successfully in certain patient populations. Yamada et al.^10^ and Herweg et al.^5^ reported the successful ablation of LV VTs via a transseptal approach and the crossing of a mechanical mitral valve prosthesis.^5^ In the latter study, a recurrence of monomorphic VT at two months later required a second VT ablation procedure using the same transseptal–transmitral approach.^5^ Transventricular septal access to the LV has been also reported in transcatheter aortic valve implantation procedures under intracardiac echocardiography (ICE) guidance.^11^ The use of ICE during transventricular septal puncture has been recommended from the viewpoint of safety; however, while it is generally used in the United States, it is not common in Europe, as health insurance agencies do not cover the costs of ICE catheters. Subcostal echocardiography was particularly useful for the confirmation of no entrapment of the tricuspid septal leaflet at the transventricular access point in the current case. Coronary angiography should be performed to assess the presence of large septal coronary artery perforators at the region of the midinterventricular septum, where safe access could be attempted.^1^ Transventricular access by subcostal echocardiographic guidance may be considered as an alternative route, particularly in critically ill patients when conventional percutaneous transaortic or transmitral valve access approaches are not possible.^12^

## Figures and Tables

**Video 1:** Subcostal echocardiography shows the transventricular crossing of a 3.5-mm irrigated-tip ablation catheter.

**Video 2:** Fluoroscopy shows the transventricular crossing of a 3.5-mm irrigated-tip ablation catheter.

**Video 3:** Fluoroscopy shows transventricular removal of a 3.5-mm irrigated-tip ablation catheter at the end of the procedure.

**Video 4:** CARTO® 3 three-dimensional electroanatomic mapping (Biosense Webster, Diamond Bar, CA, USA) shows that the low bipolar voltage (< 1.5 mV) consistent with the scar was present in both the inferolateral LV apex and where the successful point was in the middiastolic potential region.
